# Pituitary Stalk Thickening: Causes and Consequences. The Children’s Memorial Health Institute Experience and Literature Review

**DOI:** 10.3389/fendo.2022.868558

**Published:** 2022-05-20

**Authors:** Elżbieta Moszczyńska, Karolina Kunecka, Marta Baszyńska-Wilk, Marta Perek-Polnik, Dorota Majak, `Wiesława Grajkowska

**Affiliations:** ^1^Department of Endocrinology and Diabetology, The Children’s Memorial Health Institute, Warsaw, Poland; ^2^Department of Oncology, The Children’s Memorial Health Institute, Warsaw, Poland; ^3^Department of Diagnostic Imaging, The Children’s Memorial Health Institute, Warsaw, Poland; ^4^Department of Pathology, Children’s Memorial Health Institute, Warsaw, Poland

**Keywords:** pituitary stalk thickening, diabetes insipidus, germinoma, histiocytosis, lymphocytic hypophysitis, sarcoidosis, children

## Abstract

**Background:**

Pituitary stalk thickening (PST) is a rare abnormality in the pediatric population. Its etiology is heterogeneous. The aim of the study was to identify important clinical, radiological and endocrinological manifestations of patients with PST and follow the course of the disease.

**Materials and Methods:**

It is a study conducted in 23 patients (13 boys) with PST with/without central diabetes insipidus (CDI) diagnosed between 1990 and 2020 at Children’s Memorial Health Institute (CMHI) in Warsaw, Poland. We analyzed demographic data, clinical signs and symptoms, radiological findings, tumor markers, hormonal results, treatment protocols and outcomes.

**Results:**

The median age at the diagnosis of PST was 9.68 years (IQR: 7.21-12.33). The median time from the onset of the symptoms to the diagnosis was 2.17 years (IQR: 1.12-3.54). The most common initially reported manifestations were polydipsia, polyuria and nocturia (82.6%); most of the patients (56.5%) also presented decreased growth velocity. Hormonal evaluation at the onset of PST revealed: CDI (91.3%), growth hormone deficiency (GHD) (56.5%), hyperprolactinemia (39%), central hypothyroidism (34.8%), adrenal insufficiency (9%), precocious puberty (8.7%). The majority of the patients were diagnosed with germinoma (seventeen patients – 73.9%, one of them with teratoma and germinoma). Langerhans cell histiocytosis (LCH) was identified in three patients (multisystem LCH in two patients, and unifocal LCH in one patient). A single case of atypical teratoid rhabdoid tumor, suspected low-grade glioma (LGG) and lymphocytic infundibuloneurohypophysitis (LINH). The overall survival rate during the observational period was 87.0%.

**Conclusions:**

The pituitary infundibulum presents a diagnostic imaging challenge because of its small size and protean spectrum of disease processes. Germinoma should be suspected in all children with PST, especially with CDI, even when neurological and ophthalmological symptoms are absent.

## Introduction

Pituitary stalk thickening (PST) is a relatively rare abnormality in children. Its diagnosis may be challenging due to the diverse clinical picture.

There are various reasons for PST, such as neoplastic, inflammatory or infectious diseases (1). PST mainly affects teenagers and is often detected with magnetic resonance imaging (MRI) in patients with central diabetes insipidus (CDI) (2). Pituitary stalk lesions are sometimes accidentally detected during brain MRI.

In most cases, the diagnosis is based on radiological imaging and laboratory tests. Because of the risk associated with obtaining a pituitary stalk biopsy, only a small subset of patients had their diagnoses histopathologically confirmed (3, 4). In some patients, the diagnosis was made following a pathology examination of a specimen obtained from a neurosurgical procedure of removing a hypothalamic tumor. Despite the development of MR imaging techniques, PST remains a conundrum for clinicians.

The aim of the study was to analyze the clinical, radiological and endocrinological evaluation of 23 cases of patients with PST with/without CDI and follow the course of the disease.

## Materials and Methods

Our research included 23 children (10 girls, 13 boys) diagnosed between 1990 and 2020 at Children’s Memorial Health Institute (CMHI) in Warsaw, Poland. We included patients who underwent head MRI with the first radiological finding being PST. We excluded patients with massive tumor infiltration around the pituitary stalk, pituitary gland and sella turcica described in the first performed MRI. The patient’s medical data were analyzed both prospectively and retrospectively. They included patient demographics, presenting signs and symptoms, radiological findings, tumor markers, hormonal results, treatment protocols and outcome. The characteristics of the group are presented in **Table 1**.

**Table 1:** Characteristics of the group.

Patient No.	Gender	Age of first symptom (years)	Age at the diagnosis (years)	First reported symptom	Time from the first symptom to diagnosis (years)	Histopathological confirmation	PST dimensions (mm) in first MRI examination (APxT)	Posterior pituitary lobe	Final diagnosis
1.	F	5.38	7.84	CDI	5.22*	No	9 x 10	invisible	LGG**
2.	F	6.90	9.55	CDI	2.38	No	3.3 x 3.3	invisible	GCT
3.	F	2.00	2.16	CDI	0.16	No	3.5 x 3.5	invisible	M-LCH
4.	F	13.99	14.41	CDI	1.88	No	4.2 x 4.2	invisible	GCT
5.	M	13.00	13.33	CDI	1.65	No	5 x 5	visible	GCT
6.	M	6.92	8.94	CDI	4.80	Yes (post-surgical)	4 x 6	invisible	GCT
7.	F	5.92	7.03	PP (thelarche)	0.33	No	16 x 18	invisible	GCT
8.	M	11.25	12.00	CDI	0.75	No	5.5 x 6.3	invisible	M-LCH
9.	M	9.79	9.81	CDI	0.21	Yes (biopsy)***	9 x 8	invisible	GCT (recurrence)
10.	M	8.41	11.01	CDI	2.69	Yes (post-surgical)	6 x 6	invisible	GCT
11.	M	5.34	6.76	CDI+PP	1.44	Yes (post-surgical)	4 x 4	invisible	GCT
12	M	9.75	10.4	CDI	1.75	Yes (post-surgical)	3.9 x 3.9	invisible	U-LCH
13.	M	11.51	14.2	CDI	2.72	Yes (biopsy)	6 x 7	invisible	GCT
14	F	5.92	7.81	DGV	2.61	Yes (biopsy)	3 x 3	invisible	GCT
15.	F	4.97	5.44	CDI+HV	0.07	Yes (post-surgical)	11 x 12	not described	Atypical teratoid rhabdoid tumor
16.	M	6.82	7.21	CDI	2.55	Yes (post-surgical)	5 x 5	visible	GCT
17.	M	5.92	9.30	CDI	3.54	Yes (post-surgical)	10 x 7	not described	GCT
18.	F	1.97	7.16	CDI	0.14	Yes (post-surgical)	thickening	invisible	Teratoma +GCT
19.	F	9.07	11.04	SDV	2.16	Yes (post-surgical)	10 x 10	not described	GCT
20	M	7.89	12.34	DGV	4.96	Yes (post-surgical)	thickening	not described	GCT
21	F	13.40	14.88	CDI +HV	2.00*	No	4 x 4	invisible	LINH**
22	M	9.70	9.70	CDI	2.18	No	4 x 4	invisible	GCT
23	M	10.39	10.52	CDI	0.13	No	3.5 x 3.5	visible	GCT

MRI, magnetic resonance imaging; AP, anterior-posterior dimension; T, transverse dimension; CDI, symptoms of central diabetes insipidus; DGV, decreased growth velocity; PP, precocious puberty; HV, headache and vomiting; SDV, sudden deterioration in vision; GCT, germ cell tumor; LGG, low-grade glioma; M-LCH, multisystem Langerhans cell histiocytosis; U-LCH, unifocal Langerhans cell histiocytosis; LINH, lymphocytic infundobuloneurohypophysitis, *time from the first symptom, the patient has been still observed, **suspected diagnosis, the patient has been still observed, ***Tumor recurrence, a biopsy of the primary lesion.

### MRI Evaluation

In line with Godano et al., we defined the correct pituitary stalk dimensions as 2.35-2.82 mm proximally, 1.79-2.45 mm at the midpoint, and 1.28-1.78 mm distally (5). MRI scans were evaluated and described by a specialist in radiology.

### Hormonal and Other Evaluations

Growth hormone deficiency (GHD) was defined based on a growth hormone (GH) peak of less than 10 ng/ml in 3 tests (a single overnight test and two pharmacological tests – with either arginine, glucagon or clonidine).

Thyroid-stimulating hormone (TSH) deficiency was defined as decreased free thyroxine (FT4) level below the normal range accompanied by decreased levels of TSH or a normal TSH value inconsistent with the level of FT4. Adrenocorticotropin (ACTH) deficiency was diagnosed in patients with basal serum cortisol levels (8 am) and a peak cortisol level of l ACTH stimulation test (1μg i.v.) lower reference limit.

Gonadotropin deficiency was confirmed by inappropriately low serum concentrations of LH (luteinizing hormone), FSH (follicle-stimulating hormone) in the presence of low circulating concentrations of sex steroids and by insufficient gonadotropin-releasing hormone (GnRH) mediated release of LH and FSH.

CDI was diagnosed if serum osmolality was above 300 mOsm/kg and urine osmolality was below 300 mOsm/kg. A water deprivation test was also performed in some patients (6).

Tumor markers such as alpha-fetoprotein (αFP) and beta-human chorionic gonadotropin (βhCG) were measured in the blood serum and in some cases in the cerebrospinal fluid (CSF).

LH, FSH, estradiol, testosterone, prolactin (PRL), cortisol, TSH, free triiodothyronine (FT3), FT4, antithyroglobulin antibody (anti-Tg), thyroid peroxidase antibody (anti-TPO) were measured with the chemiluminescent immunoassay method (Alinity i/Abbot).

GH, insulin-like growth factor-1 (IGF-1) and αFP were measured with chemiluminescent immunoassay (IDS-iSYS/IDS). βhCG and ACTH were measured with the chemiluminescent immunoassay method (Cobas e411/Roche).

### Additional Data

Growth velocity was assessed based on the percentage grid. Anthropometric data were collected retrospectively from the patients’ medical records and prospectively during the hospitalization or ambulatory visit.

Some patients underwent a lumbar puncture. Basic laboratory parameters including protein and glucose levels in the CSF were measured. Additionally, microscopic analysis for the presence of cancer cells in the CSF was performed. QuantiFERON-TB test was performed to rule out tuberculosis. In selected cases, angiotensin-converting enzyme (ACE), and antibodies against the pituitary gland and anti-hypothalamus were measured. Some patients underwent additional chest imaging as a part of the differential diagnosis: X-ray or high-resolution computed tomography (HRCT). Bone scintigraphy and ^18^F-fluorodeoxyglucose positron emission tomography/computed tomography (^18^F-FDG PET/CT) were performed in selected patients.

In specific cases, pituitary stalk biopsy was performed.

### Overall Survival

Overall survival (OS) was defined as the time between diagnosis and death due to any cause or last follow up contact for patients who were alive.

### Statistics

Statistical analyses were performed using Stata^®^ Software ver. 14.1 (StataCorp LLC). Nominal parameters were presented as a percentage frequency. The Shapiro-Wilk test was used to test the univariate normality assumption of continuous variables. The Kaplan-Meier survival curves were used to assess the overall survival of the study population.

## Results

### Demographics

Median follow-up was 6.58 years (95% CI 2,60- 10,17). At the moment of PST diagnosis the age was variable: the youngest child was 2 years and 2 months old and the oldest one was 14 years and 11 months old (median age 9.68 years, IQR: 7.21-12.33). In the group with GCTs (germ cell tumors) the median age was 9.81 years (IQR 7.81-11.15).

The median age when the first symptoms occurred was 7.89 (IQR: 5.92-10.39) years, in the group with GCTs it was 7.89 years (IQR: 5.92-9.79). The time from the onset of the first symptoms to the final diagnosis also diversified. The median time was 2.17 years (IQR: 1.12-3.54) and 2.69 years (IQR: 1.65-3.54) in all and germinoma patients, respectively. In seven cases, the diagnosis was made within one year, four more children were diagnosed within two years. Conversely, three patients had been observed for more than 4 years before the final diagnosis. One had been observed for 5.22 years without receiving the final diagnosis (**Table 1**).

### Clinical Findings

The signs of CDI were most common indication for further diagnostics: polydipsia, polyuria or nocturia reported by sixteen patients (69.6%). Additionally, in three patients (13%) the first reported symptom was CDI and it was accompanied by precocious puberty (in one patient) and headache (in two patients).

However, when analyzing the patients’ growth charts we found that decreased growth velocity (DGV) ahead of CDI and a sudden deterioration in vision (SDV) lasting for up to 4 years were actually the first symptoms in nine cases in this group.

At onset thirteen patients (56.5%) presented DGV (three patients with short stature), nine patients (39.2%) had headaches, and in three of them (13.0%) vomiting was also noted. Moreover, the patients reported vision disorders like strabismus or double vision (four patients, 17.4%), weight loss (three patients, 13.0%), fatigue (three patients, 13.0%), nodular lesions on the skin (two patients, 8.7%), hearing disorders (one patient, 4.3%), somnolence (one patient, 4.3%), limb numbness (one patient, 4.3%) and increased appetite (one patient, 4.3%). Breast development was the first symptom in a 7-year-old girl.

As regards patient No. 9 PST was found in a routine MRI of the head performed 2 months after treatment for germinoma of the pineal gland. This patient had also developed CDI, decreased appetite and loss of weight 2 months before the final diagnosis.

### Hormonal Results

CDI was the main reason for further diagnostics in nineteen patients (82.6%). In another two cases (8.7%) CDI was diagnosed during the first hospitalization in the Endocrinology Department. GH tests were performed in thirteen children (56.5%) with DGV. GHD was found in all of them. IGF-1 was also decreased in this group. Based on laboratory tests, central hypothyroidism was found in eight children (34.8%). Adrenal insufficiency was diagnosed in two per twenty two tested (9.0%) patients, and incomplete adrenal reserve was confirmed in 3 children (13.0%) after a stimulation test with an ACTH analogue. PRL serum concentration was increased in nine patients (39%). The maximum level of PRL was 109.4 ng/ml and the mean of the elevated values was 50.2 ng/ml. Precocious puberty (PP) was found in two children (8.7%; a 7-year-old girl and a boy aged 6 years and 10 months). In both cases, elevated levels of βhCG were present.

The results of hormone tests are summarized in **Table 2**.

**Table 2:** Hormonal and other evaluations.

Patient No.	TSH(N:0,4-5,0 uIU/ml)	FT4(N:0,6-1,4 ng/dl)	Morning cortisol (N:5-20μg/dl)	PRL M(N:3,46-19,4 ng/ml) F(N:5,18-26,53ng/ml)	IGF-1(ng/ml)	αFP (IU/ml)		βhCG (mIU/ml)	
						Serum	CSF	Serum	CSF
1.	3.146	0.87	18.7	54.95	56 (N:67.2-349.4)	2.85 (N<5)	2.01 (N<5)	<0.03 (N<0.1)	0.03 (N<0.1)
2.	1.679	0.87	10.8	18.1	56 (N: 59-297)	3,28 (N<5)	2.19 (N<5)	<0.03 (N<0.1)	**0.18** (N<0.1)
3.	3.611	1.05	11.7	5.29	74.6 (N:59-297)	– –	– –	– –	– –
4.	2.978	0.67 (N:0.8-1.8)	10.6	27,8	157 (N:214-753)	2,67 (N<5)	2,46(N<5)	<0.03 (N<0.1)	**↑** 0.06 **- 0.37** (N<0.1)
5.	1.74	1.3	11.2	3.9	99.2 (N:96.9-406.6)	**71.6** (N<5)	–	**0.16** (N<0.1)	–
6.	1.84	1.11	21.4	10.9	37.3 (N:128-458)	2.65 (N<5)	2.32 (N<5)	<0.03 (N<0.1)	0.06 (N<0.1)
7.	1.6	0.48	–	37	69 (N:89-345)	**44.6**(N<5)	–	**27.0** (N<3.9)	–
8.	1.38	0.8	19.1	9.08	64 (N:115-498)	2.51 (N<5)	–	<0.03 (N<0.1)	–
9.	3.73	0.94	16.1	28	–	1,8 (N:0.89-8.78)	–	↑ 0,04 **- 0.48** (N<0.1)	–
10.	2,56	0.8 (N:0.97-1.67)	3.2	109.4	60.4 (N:68-388)	<1.3 ng/ml (N:0.0-8.0)	2.07 ng/ml (N:0.0-8.0)	**2.0** (N<0.1)	0.09 (N<0.1)
11.	0.656	1.2	19.7	11.32	–	2.7 ng/ml (N<5)	–	**30.1** (N<0.1)	<0.03 (N<0.1)
12.	2.5	0.61	5.8	11	170 (N:83.6-361.6)	1.6 ng/ml (N<10.9)	–	0 (N<2.5)	–
13.	2.82	0.73 (N:0.8-1.8)	14.3	60.7	101 (N:139-727)	1.8 (N:0.89-8.78)	–	<0.03 (N<0.1)	–
14.	2.63	0.58 (N:0.84-1.47)	1.8	42.9	27.5 (N:80-395)	**5.33** (N<5)	2.35 (N<5)	0.03 (N<0.1)	0.08 (N<0.1)
15.	0.211	0.52	5.7	8.4	–	1.7 (N<5)	–	<3.5 (N<3.5)	–
16.	1.1	1.5	26.3	18	0,3U/ml (wnr)	1.9 (N<5)	–	<0.2 ng/ml (wnr)	–
17.	2.133	0.83	10.06	–	48 (N:83.6-361.6)	–	–	–	–
18.	2.27	1.3	16.5	10.3	24.43 (N:42.0-240.4)	1.5 (N<5)	–	3.9 (wnr)	–
19.	2.9	0.67	10,63	12.8	92 (N:92.6-452.6)	4.7 (N<5)	–	0.8 ng/ml (N<1)	–
20.	0.40	0.46	7.25	46.4	53 (N:147-551)	2.9 (N<10)	<1 (N<10)	0.4 ng/ml (N<1)	0.2 ng/ml (N<1)
21.	1.49	1.12	12.8	19.0	–	2.66 (N<5)	–	<0.03 (N<0.1)	–
22.	1.91	0.5	13.5	44.3	73 (N:96.9-406.6)	2.5 (N:0.89 -8.78)	<0.03 (N:0,89-8,78)	0.04 (N<0.1)	<0.03 (N<0.1)
23.	1.24	0.85	13.9	–	112 ng/ml (N:85-553)	4.06 (N<5)	2.03 (N<5)	0.04 (N<0,1)	**0.16** (N<0.1)

TSH, thyroid-stimulating hormone; FT4, free thyroxine; PRL, prolactin; αFP- alpha-fetoprotein; βHCG, beta-human chorionic gonadotropin; IGF-1, insulin-like growth factor 1; N- normal range; wnr, within normal range; M- male; F- female; **↑ -** increased level in subsequent tests. Bold type - increase the level of βhCG and αFP.

### MRI Characteristics

**Figure 1:**
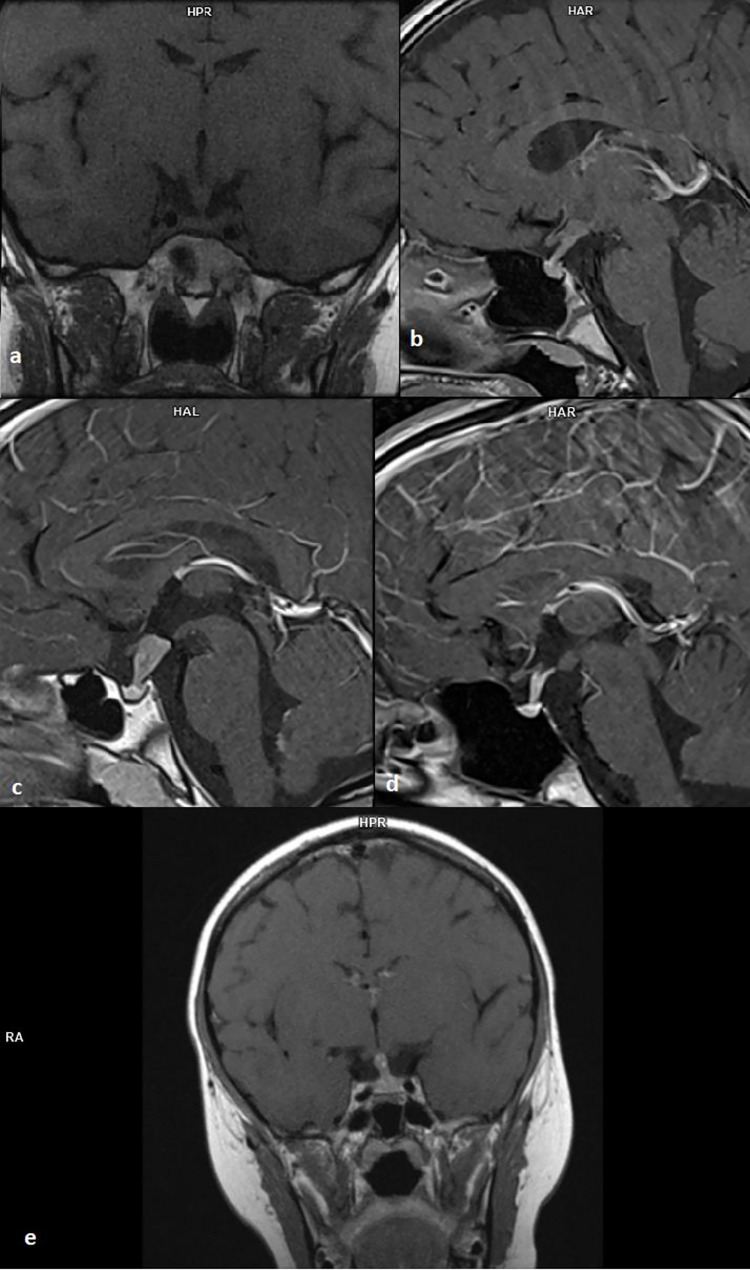
**(A)** 12-year-old patient with multisystem Langerhans cell histiocytosis, T1 sequence, coronal projection; **(B)** 9-year-old girl with germinoma, T1 sequence, sagittal projection; **(C)** 9-year-old boy with recurrence germinoma, T1 sequence, sagittal projection; **(D)** 14-year-old girl with germinoma, T1 sequence, sagittal projection; **(E)** 8-year-old boy with germinoma, T1 sequence, coronal projection.

Mean pituitary stalk dimensions in the first MRI were: AP 6.18+/-3.3 mm (min. 3.0 mm, max. 16 mm), transverse 6.37+/-3.56 mm (min. 3.0 mm, max. 18 mm) (**Figure 1**).

The posterior lobe of the pituitary gland was not visible in sixteen cases (69.6%). Almost all of those patients presented the clinical signs of CDI (93.7%). Normal posterior lobe of the pituitary was visualized in a typical location only in three patients (13%). All of them were diagnosed with CDI. In four cases (17.3%) the posterior lobe of the pituitary gland was not described by the radiologist.

The progression of the disease was differentiated in subsequent MRIs. The velocity of tumor growth varied from 1.5 to 3.7 mm within the first 6 months of observation. Focal lesions in the pituitary gland were found during follow-up in three patients (13%). The infiltration of the hypothalamus was observed in another ten cases (43.5%). In one case, the lesion enlarged and infiltrated the pontocerebellar angle reservoir, the left internal jugular bulb, the frontal horns of the lateral ventricles, the internal auditory canal, n. IX and X on the right within 2 months. Conversely, one patient developed a spontaneous involution of the lesion within 2 years of follow-up.

### Additional Tests

Serum αFP was increased in three cases finally diagnosed with germinoma. Serum βhCG was increased in five patients who were also diagnosed with germinoma. One of those patients had an initial normal level of βhCG, then an elevated concentration during the follow-up (0.04 mIU/ml to 0.48 mIU/ml, n<0.1 mIU/ml) (**Table 2**).

Nine patients underwent QuantiFERON test. None of them had positive results. In three patients (per four tested) antibodies against the pituitary gland and anti-hypothalamus were present. One patient was diagnosed with GCT, the second with suspicion of lymphocytic infundibuloneurohypophysitis (LINH), another is still under observation for low-grade glioma (LGG).

Three patients (per six tested) had an increased ACE activity [44.1 IU/l (N:1.2-8.52), and 109 IU/l (N:12-68), 56.7 IU/l (8-52)]. Two patients were finally diagnosed with germinoma and in one LGG was suspected. As regards patient No. 4, ACE in the CSF was tested with negative results.

Both tested patients had normal vasopressin concentrations. Immunoglobulin concentrations were within the normal range among the remaining patients except patient No. 8, who had a clinically irrelevant, slightly decreased immunoglobulin M (IgM) level and an increased immunoglobulin A (IgA) level.

### Imaging Examination

Several patients underwent chest imaging (X-ray or HRCT). Consolidations in the lungs corresponding to histiocytosis were detected in two of them. Two patients had an ^18^F-FDG PET/CT – active bone, occipital and cervical lymph node lesions indicating malignancy were found in one patient. Three patients underwent Technetium-99m bone scintigraphy. The examination revealed typical lesions in two of them (with multifocal histiocytosis). As regards the first patient, the lesions were located in the skull bones, left humerus bone, and in the second patient – in the skull bones, right humerus, left scapula, ribs and vertebrae. Due to the suspicion of sarcoidosis, gallium-67 scintigraphy was performed in one patient (the result was negative).

### Cerebrospinal Fluid Analysis

Ten patients (43.4%) underwent a lumbar puncture. None of the patients had neoplastic cells in the CSF. Polymerase chain reaction (PCR) Mycobacterium tuberculosis complex tests were negative. Slightly increased βhCG was found in the CSF of three patients (up to 0.16 mIU/mL, 0.18 mIU/mL and 0.37 mIU/mL, N<0.1 mIU/mL) as opposed to not serum βhCG which was not elevated (0.04 mIU/mL, <0.03 mIU/mL and <0.03 mIU/mL, respectively, N<0.1 mIU/mL). Initially, the concentration of βhCG was within the normal range in one of those patients, but in subsequent tests, an increased level of βhCG (from 0.06 mIU/mL to 0.37 mIU/mL, N<0.1 mIU/mL) was observed. The concentration of αFP (measured in 9 patients) was normal (**Table 2**).

A higher activity of macrophages with the atypical expression of CD14 was found in one case. Another case was characterized by an increased number of neutrophils and lymphocytes in the CSF. ACE concentration was measured in the CSF of one patient and appeared to be within the normal range.

### Histological Findings

Two patients (8.7%) underwent pituitary stalk biopsy and were both diagnosed with germinoma. (**Figures 2.1A–C**) A biopsy of the primary lesion was performed in one patient with tumor recurrence (4.3%).

**Figure 2:**
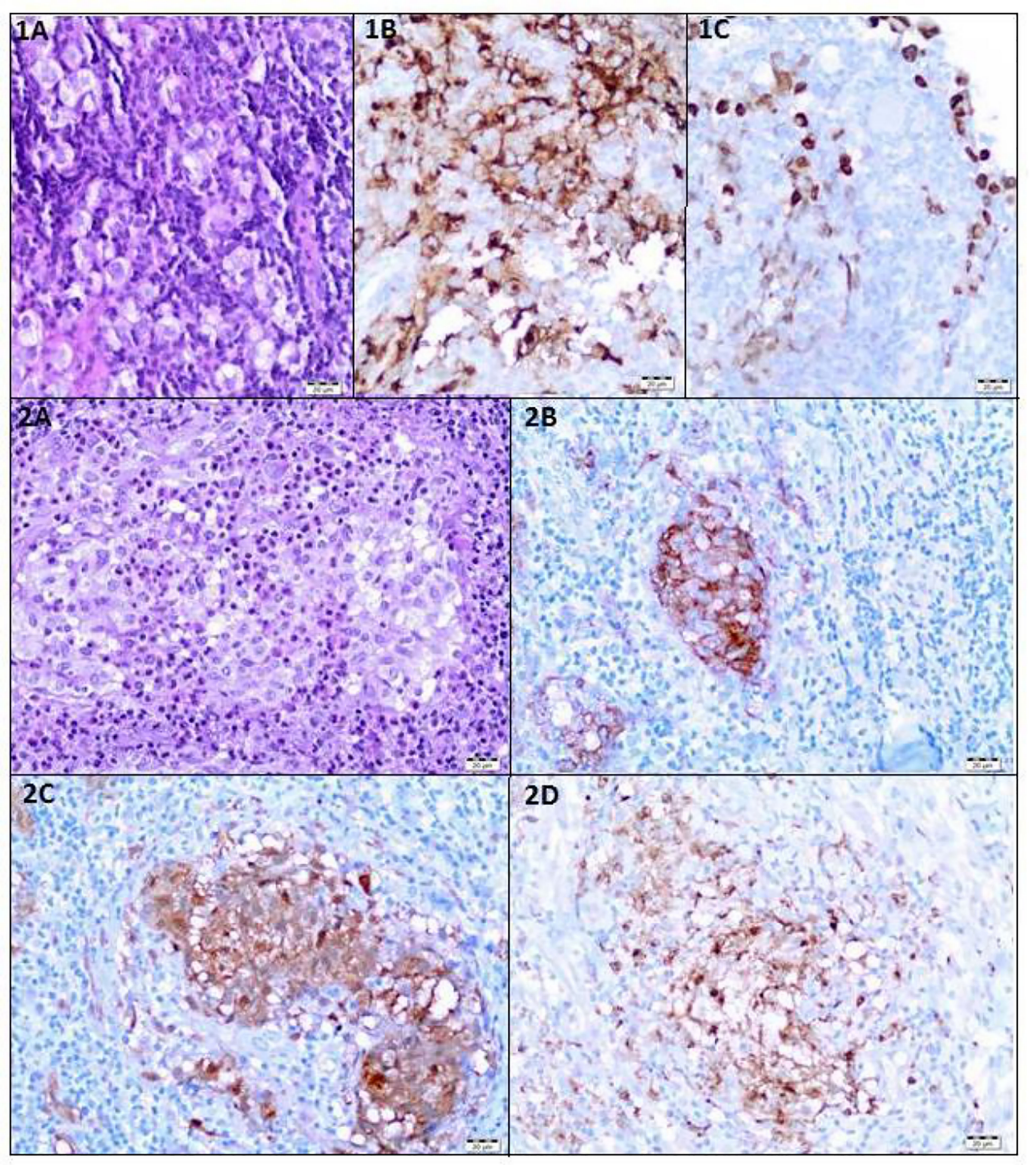
Histological Findings. **(1A–C)** Patient 14 - Germinoma. IA. Large, polygonal cells with abundant clear cytoplasm. Hematoxylin and Eosin staining (HE). **(1B)**. Immunoexpression of PLAP in neoplastic cells. **(1C)** Immunoexpression of Oct3/4 in neoplastic cells. **(2A–D)** Patient 12 - Langerhans cell histiocytosis. **(2A)** Langerhans cells with numerous eosinophils. HE staining. **(2B)** Immunoexpression of CD1a in neoplastic cells. **(2C)** Immunoexpression of S100 in neoplastic cells. **(2D)** The expression of CD68 in neoplastic cells.

One patient suspected of multisystem Langerhans cell histiocytosis (M-LCH) underwent skin, skull lesion, lymph node, and bone marrow biopsies with the results being inconclusive. The unifocal form of LCH (U-LCH) was diagnosed based on the histopathological examination of the lesion of the infundibulum (**Figures 2.2A–D**).

Ten patients (43.5%) underwent a surgery, and the diagnosis was based on postoperative histological examination.

Ten patients (43.5%) were treated without histopathological confirmation. The diagnosis was based on typical signs and symptoms, laboratory tests (oncoprotein) and typical MRI images – six patients were suspected to have germinoma, one – LGG, one – LINH and two – histiocytosis.

### Etiological Spectrum

**Figure 3:**
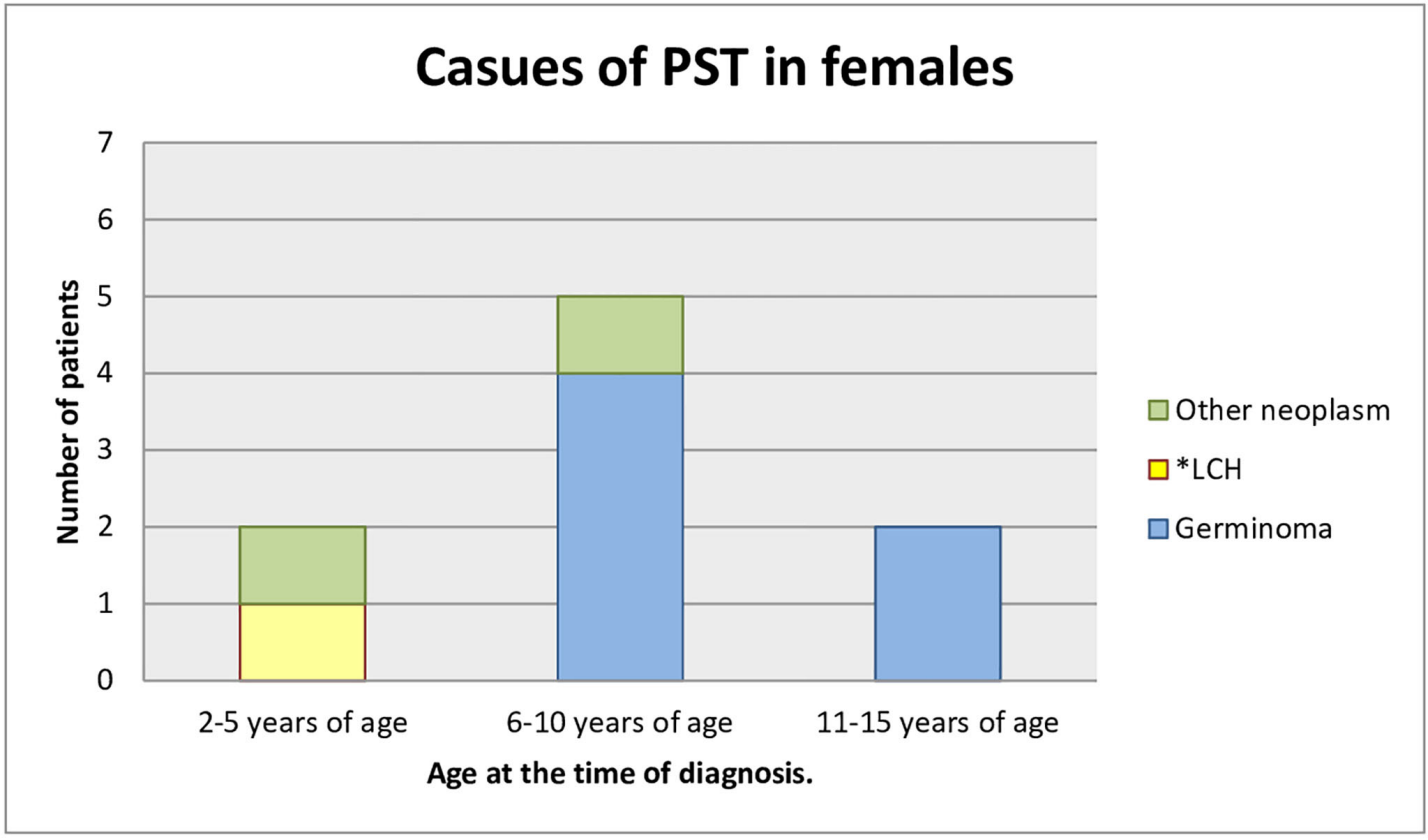
Causes of PST in females (age at the time of the diagnosis). *LCH, Langerhans cell histiocytosis.

**Figure 4:**
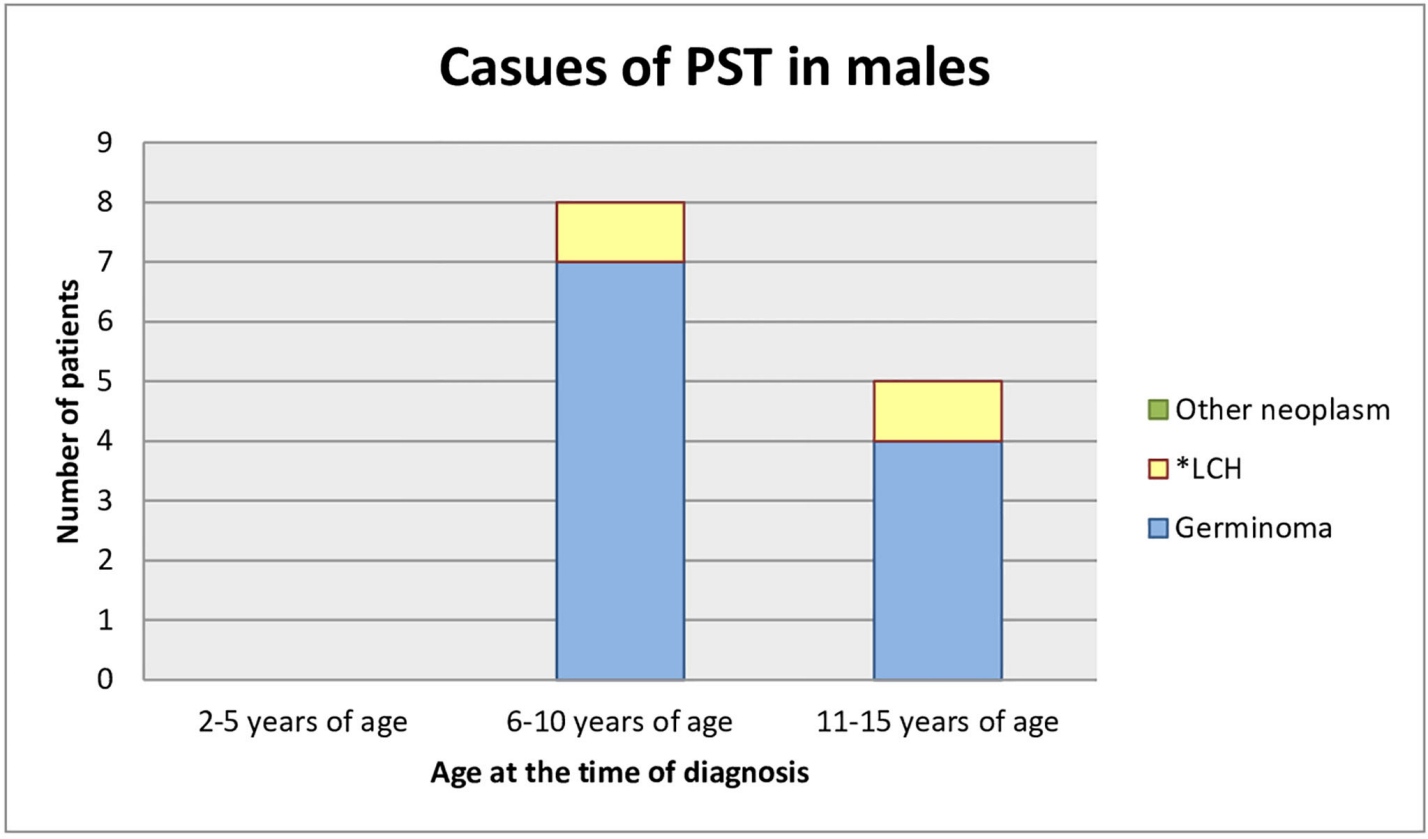
Causes of PST in male (age at the time of the diagnosis). *LCH, Langerhans cell histiocytosis.

Most of the patients who had already been diagnosed developed neoplastic diseases. Sixteen children (69.6%) were diagnosed with germinoma. One of them had a pituitary stalk metastasis of germinoma primarily located in the pineal gland. One (4.3%) child had a mixed tumor of germinoma and teratoma and there was one case (4.3%) of atypical teratoid rhabdoid tumor. One patient (4.3%) was under observation for a low-grade glioma, remained untreated, and the tumor decreased in size in subsequent MR scans. Three children (13.0%) developed histiocytosis. The pituitary stalk lesion regressed within 2 years of follow-up in one patient (4.3%), which may indicate lymphocytic inflammation (**Figures 3**, **4**).

### Management

Ten children (43.4%) underwent surgical treatment. Nine of them underwent subsequent radiotherapy and seven – chemotherapy. Five children (21.7%) were treated with primary radio-chemotherapy. The mean irradiation dose was 3600 cGy for the tumor and 5400 cGy for the whole CNS. Chemotherapeutic agents used in germ cell tumors included cyclophosphamide, cisplatin, carboplatin, bleomycin, and etoposide. Four children (17.4%) underwent only chemotherapy. Methotrexate, vinblastine and steroids were mainly used in patients with histiocytosis. One patient required chronic treatment with methotrexate due to recurrent histiocytosis.

### Prognosis and Outcomes

Tumor and oncological treatment had long-term consequences for all the patients. The most common complication was multi-hormonal pituitary insufficiency. All the children who developed CDI required desmopressin (before the treatment two of them had not presented the signs of CDI). Vasopressin deficiency was transient in two cases and the treatment was discontinued after 6 months and two years, respectively. The remaining patients (21/23, 91.3%) required a steady supply of desmopressin at a variety of doses: from 7.5 μg to 180 μg per day.

Sixteen patients (69.6%) developed secondary hypothyroidism and required supplementation of L-thyroxine in doses between 37.5 μg and 150 μg per day.

ACTH deficiency was identified in fifteen patients (65.2%) and hydrocortisone replacement therapy was introduced at daily doses of 10 to 25 mg.

Eleven patients (47.8%) were diagnosed with GHD and they were treated with recombinant human growth hormone (rhGH), doses 0.24 to 0.65 units/kg/week. Nine patients (81.8% of the treated group) achieved the height consistent with parental prediction between the 10^th^ and 90^th^ percentile.

In eleven cases (47.8%) we observed delayed puberty associated with hypopituitarism in terms of LH and FSH and damage to the gonads in the course of radio- or chemotherapy. So far, puberty had occurred on time only in three cases (13.0%). Six patients (26.1%) were still in the pre-pubertal phase. Six male patients received testosterone supplementation. Two patients (8.7%, also the one with PP at the onset), required hormone replacement therapy with estradiol and norgestrel after the oncological treatment.

Metabolic disorders were also observed in the patients. During the follow-up until 18 years of age, seven patients (30.4%) developed obesity, three patients (13.0%) had insulin resistance and three patients (13.0%) developed non-alcoholic steatohepatitis. One patient (4.3%) developed osteoporosis.

Neurologically, three patients (13.0%) experienced partial hearing loss, two patients (8.7%) had a reduced visual field and another two patients (8.7%) developed epilepsy. Patient No. 4 required chronic methotrexate treatment for recurrent histiocytosis.

### Adult Life

In order to find out how patients functioned in adulthood after the end of treatment, a survey was conducted in former patients. Information was collected by phone and by sending questionnaire forms by post. Contact was made with seven out of nine (77.8%) adult patients. The remaining two patients died during the observational period. The median age at the moment of contact with the adult patients was 22.56 years (IQR: 22.25-38.03). The median follow-up was 15.12 years (95% CI: 9.93 - 26.81).

The patients were under multidisciplinary care: endocrinology, diabetes, neurology, ophthalmology, gynecology. One of the patients, who lived abroad, remained only under the care of a general practitioner. One patient was under the care of a psychiatrist for obsessive-compulsive disorder.

Five of them (71.4%) required hydrocortisone supplementation (15 to 30 mg per day), L-thyroxine (100 to 200 μg per day) and antidiuretic hormone analogue (30 to 540 μg per day). Three male patients required testosterone supplementation (100 to 200 mg per 2 weeks). Two female patients required hormone replacement therapy.

Four patients had ophthalmic complications (57.1%): drooping eyelid, reduced visual acuity, and one had eye amblyopia. Deafness persisted in two patients (28.6%).

Metabolic syndrome was the most common complication in this group. Four patients (57.1%) were obese (BMI 31.6-52.5 kg/m2), one (14.3%) was overweight. Two patients (28.6%) presented with lipid disorders, three (42.9%) had type 2 diabetes (T2D) treated with oral hypoglycemic medications, two (28.6%) had hypertension.

Three patients (42.9%) graduated from secondary school. One patient (14.3%) completed tertiary education. Two of them (28.6%) were studying at a university. None of them had a permanent partner or offspring.

### Mortality

Two patients died after the treatment of GCTs and one after atypical teratoid rhabdoid tumor treatment.

Patient No. 5 died at the age of 21 due to the progression of neoplastic disease – metastatic spread to the CNS, eight years after the diagnosis of GCTs.

Patient No. 15 developed acute myeloid leukemia five years after the oncological treatment of atypical teratoid rhabdoid tumor (surgery, radio-chemotherapy) and died three months later, at the age of 12.

Patient No. 16 died for an unknown reason, twenty years after the treatment of GCTs at the age of 30.

**Figure 5:**
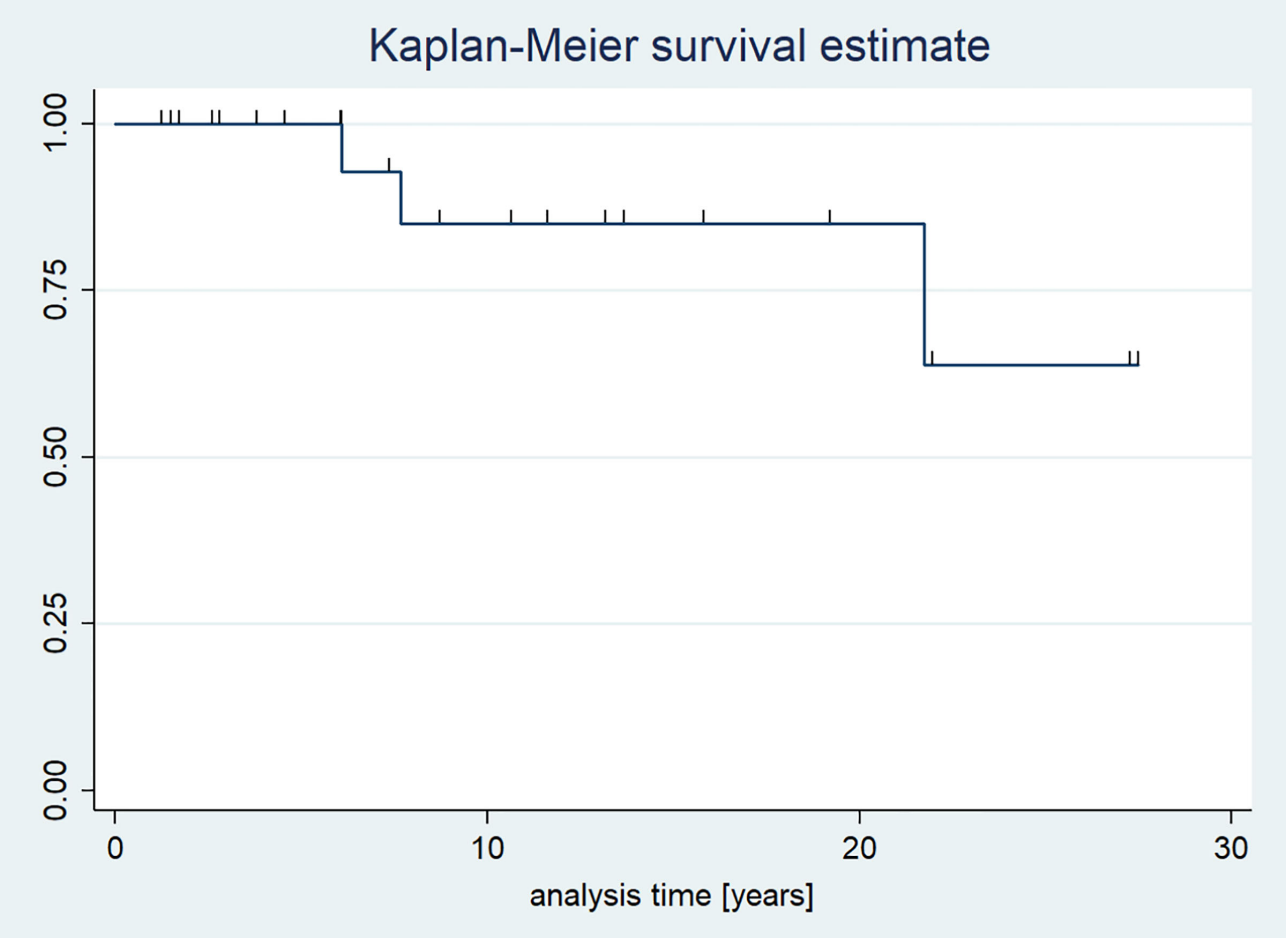
Kaplan-Meier survival curves of patients with pituitary stalk thickening.

The 5-year survival rate was 100%, and the 10-year survival rate was 91.3%. The overall survival rate during the observational period was 87.0%. The median overall survival was not reached (**Figure 5**).

**Figure 6:**
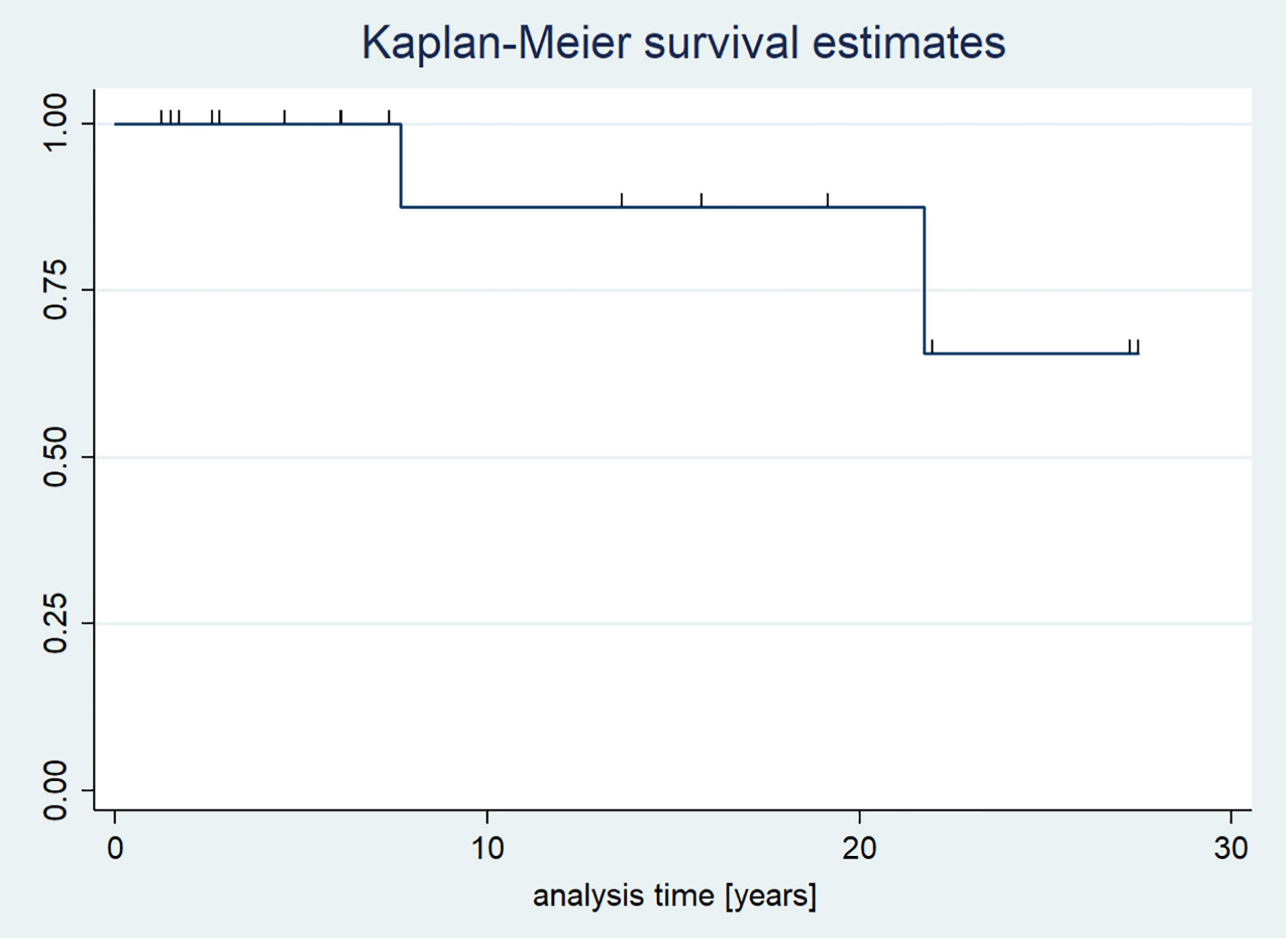
Kaplan-Meier survival curves in children with GCTs.

As regards children diagnosed with GCT, the overall survival rate during the observational period was 88.2%. Two patients died (**Figure 6**).

## Discussion

The present study includes a retrospective analysis of the clinical, anthropometric, hormonal, and radiological aspects of 23 children with PST.

The actual incidence of pituitary stalk lesions in the general population remains unknown, but it is a rare condition. Previous studies determined the correct pituitary stalk dimension as 3.25+/-0.56 mm in the transverse diameter at the optic chiasm and 1.91+/-0.40 mm at its pituitary insertion (7). The only pediatric study including 102 children aged 7-12 years, demonstrated smaller stalk sizes (2.35-2.82 mm proximally, 1.79-2.45 mm at the midpoint, and 1.28-1.78 mm distally) on T1 and T3-DRIVE images than other adult studies did (5). We also qualified patients for the study based on this definition. Some authors suggested the following classification of PST: mild (3-3.9 mm), moderate (4-6.5 mm) or severe (>6.5 mm) PST (8).

Numerous studies confirmed that CDI was the most common initial symptom of PST (2, 4, 9). Every pediatric patient with CDI should undergo MRI imaging of the pituitary hypothalamic axis to confirm the diagnosis and perhaps determine the etiology (10).

In most of our patients, endocrine disorders, especially CDI, were the reason to start diagnostics and perform the MRI of the head. CDI was an indicator to continue diagnostics in 82.6% of patients. Anterior pituitary hormone deficiencies were present in 56% of children.

Researchers confirmed that children with PST and CDI were likely to develop a neoplastic process (11, 12). Anterior pituitary hormone dysfunction and the enlargement of lesions over time are predictive of neoplasia (11).

According to Devuyst et al. young age, male gender, a very large PST, a higher number of anterior pituitary deficits as well as the serum level of PRL above 1.3 times the upper limit of normal are all non-invasive parameters increasing the likelihood of a neoplastic origin of patients with CDI and PST (13). Furthermore, the findings by Catli et al. suggested that accompanying anterior pituitary hormone deficiencies and decreased growth velocity at follow-up in patients with CDI might be considered as indicators of the organic etiology (14). Another study revealed that older age at diagnosis (over five years of age), short stature and anterior pituitary involvement were indicative of the organic etiology of CDI (15).

PST in patients with CDI necessitates differential diagnosis that includes germinoma, LCH, lymphocytic hypophysitis, neurosarcoidosis, tuberculosis and adenoneurohypophysitis of another category, infiltration from adjacent neoplasms, metastasis, and a congenital lesion (16, 17). In children under ten years of age, congenital disorders and neoplasms remain prevalent (18). Infectious/inflammatory disorders prevail in children over 11 (19). Neoplastic infiltration and metastasis are radiographically distinguishable from regional and systemic findings. Diagnostic information for neurosarcoidosis and tuberculosis will be provided with chest x-ray studies and immunological and serological examinations. Infundibulohypophysis may be distinguished from occult germinoma by the presence of an enhanced pituitary lesion (16). However, LCH may take a course quite similar to occult germinoma. The MRI findings of hypophyseal histiocytosis also demonstrate a homogeneously enhanced, thickened stalk and absent posterior pituitary hyperintensity (20). Systemic bone lesions should be searched, but in cases of solitary hypophyseal lesions, obtaining a biopsy sample is mandatory.

A definitive diagnosis is needed, because these diseases require different methods of treatment and are associated with different prognoses. The differential diagnosis between the diseases using tumor markers and radiological examinations is often difficult, and only histopathological examination leads to a definitive diagnosis.

Previous studies analyzing the pediatric population showed that most patients with PST and CDI were diagnosed with neoplastic disorders (38-75%) (3, 4, 9). According to the literature, the prevalence of GCT in children as a cause of PST was estimated at 10-25% (**Table 3**). A higher percentage of GCTs (43.7%) in a cohort from Taiwan was one exception, but it was consistent with the higher rates of GCTs described in some Asian populations (24).

**Table 3:** Prevalence of eventual causes responsible for PST (with/without CDI) in 8 pediatric studies and author’s series.

Study	Patients’ group	Diagnosis			
		Neoplastic disorder		Inflammatory or autoimmune	Idiopathic
		GCTs/other neoplasm	LCH		
Hamilton et al. (9) 2007, n=21, <21y		10%	19%	0%	0%
Cerbone et al. (21) 2016, n=53, <19y	CDI n=38	GCTs, Craniopharyngiomas, Optic gliomas: 27.9%	5.6%	–	–
	PST n=10	0%	0%	–	–
	PST + CDI n=5	GCTs, Craniopharyngiomas, Optic gliomas: 26.9%	38.5%	–	–
Werney et al. (12) 2015, n=10, <24.4y	PST+CDI	GCTs: 10%	30%	0%	60%
Di Iorgi et al. (22) 2014, n=40, <12.6y	PST+CDI	GCTs: 0%, Hodgkin lymphoma: 2.5%	7.5%	90%	0%
Leger et al. (18) 1999, n=26, <18 y	PST+CDI	GCTs 15.3%	19.2%	0%	65.5%
Maghnie et al. (23) 2000, n=29, <24.8y	PST+CDI	GCTs 17.2%	17.2%	3.5%	62.1%
Liu et al. (24) 2013, n=16, <17.4y	PST+CDI	GCTs 43.7%	56.2%	0%	0%
Robison et al. (11) 2013, n= 42, <19.7y	PST+CDI n=16	GCTs 25%, Lymphoma 6.25%	18.75%	18.75%	31.25%
	PST n=24	Craniopharyngioma 4.1%	0%	0%	96%
Author’s series, n=23, <14.8y	PST+CDI n=21	GCTs 71.4% (including 4.8% GCTs + teratoma) atypical teratoid rhabdoid tumor 4.8% LGG 4.8%	14.2%	4.8%	0%
	PST n=2	GCTs 100%	0%	0%	0%

LCH, Langerhans’cell histiocytosis; GCTs, germ cell tumors; CDI, central diabetes insipidus; PST, pituitary stalk thickening; LGG, low-grade glioma; n, number of patients; y, years of age.

Similarly to the literature, in our study the majority (82.6%) of patients with PST were diagnosed with neoplastic diseases, including GCTs, LGG and atypical teratoid rhabdoid tumor. Three patients had LCH and one case was observed for LINH. There were no cases of idiopathic or congenital causes of PST. Our hospital is a multidisciplinary tertiary referral center specialized in pituitary tumor treatment, which could be the reason for the prevalence of neoplastic cases (**Table 3**).

### Neurohypophyseal Germinoma and Other Neoplasms

Germinoma was diagnosed in seventeen (73.9%) patients (teratoma-germinoma in one). A metastasis to the peduncle or non-simultaneous appearance of the second germinoma location (with the first one being in the pineal gland) were identified in one case. Metastases to the pituitary stalk were rarely reported, mostly in adults, and the most common pituitary tumors were breast and lung carcinoma (3).

Another neoplastic cause of PST in our study was an atypical teratoid rhabdoid tumor. The diagnosis was made after a post-surgery biopsy in a five-year-old girl.

Based on the clinical presentation, laboratory and imaging tests LGG was suspected in one, currently an 11 years and 9 months old patient. Biopsy was not performed.

According to the 2021 World Health Organization (WHO) Classification of Tumours of the Central Nervous System the types of GCTs are as follows: germinoma, embryonal carcinoma, yolk sac tumor, choriocarcinoma, teratoma (mature, immature), teratoma with somatic-type malignancy, and mixed germ cell tumor (25). Germinoma accounts for more than half of CNS GCTs in childhood.

Some central nervous system GCTs produce tumor markers that are helpful in indicating the histological subtypes. Typically, choriocarcinoma produces βhCG, and yolk sac tumor produces αFP. Germinoma with syncytiotrophoblastic giant cells also produces human chorionic gonadotropin, but the serum titer of germinoma with syncytiotrophoblastic giant cells is usually lower than in choriocarcinoma. Mixed germ cell tumors, according to the histological elements they contain, produce various amounts of tumor markers, which may be detected both in the serum and CSF (26).

The incidence of GCTs in the United States is 0.1 per person-years, and 0.4-0.5 per 100 000 children per year in Taiwan, the median patient age is in young adolescence, usually 10-14 years (27).

The suprasellar region within the infundibulum or the pituitary stalk (30%) is the second most common site after the pineal location (45%) (28). Neurohypophyseal germinomas usually cause CDI and adenohypophysis deficiency, particularly GH secretion followed by TSH, LH, FSH secretion, with ACTH being less common (29). According to Change et al., who described 49 children with intracranial pure germinoma in Taiwan, growth hormone deficiency or low IGF-1 was diagnosed in 85.7%, adrenal insufficiency in 77.8%, CDI in 55.1%, central hypothyroidism in 48.4%, and hypogonadotropic hypogonadism in 44.4% of children (30).

CDI is the most common initial symptom of neurohypophyseal GCTs. Occasionally, CDI precedes the emergence of radiological abnormalities that indicate neurohypophyseal germinoma by several months or years (one month – 6 years). Germinoma causing CDI was termed occult germinoma and it may result in a critical delay in the accurate diagnosis and, therefore, treatment of disease (31, 32). Germinomas have an almost exclusive predominance as the occult cause of CDI in teenagers (33). CDI, symptoms of adenohypophysis deficiency (over 90%) predominate in children with the suprasellar location, with visual disturbances and increased intracranial pressure also being noted.

In addition to hypopituitarism, any tumor affecting the neurohypophyseal or pineal region and producing βhCG may cause pseudoprecocious puberty in boys and, occasionally, in girls (34–36).

βhCG is structurally similar to LH. It was found to contribute to increased testosterone production leading to peripheral precocious puberty. Reduced FSH levels may explain the lack of correlation between testicular growth and other sexual characteristics, since testicular maturation depends upon this hormone. The postulated mechanisms of precocity in females are low FSH activity of very high hCG and the high aromatase activity of the tumor (35, 37, 38). Our research revealed precocious puberty in a nearly 7-year-old boy with βhCG 30.1 mIU/ml. The symptoms of peripheral precocious puberty were observed at the age of six (Patient No. 12).

However, extremely rare cases of hCG-dependent precocious puberty were also reported in girls (35, 36). We observed only one case of precocious puberty in a 7-year-old girl (premature thelarche at 6 years of age) and serum βhCG 27 mIU/ml (Patient No. 7).

Other authors (Starzyk et al. and Kitanka et al.) also described five- and six-year-old girls with gonadotropin-releasing hormone-independent (Gn-RH-independent) precocious puberty caused by suprasellar germ cell tumor secreting βhCG and αFP and suprasellar immature teratoma secreting βhCG, respectively (35, 36).

Tumor markers like βhCG and αFP in the serum are the first-line investigation in patients with PST. Elevated levels of βhCG, αFP and/or human placental alkaline phosphatase (PLAP) should greatly enhance the suspicion of the disease (39).

Our study revealed an increased level of βhCG in the serum or CSF in 8 out of 17 patients diagnosed with germinoma (47%), and an increased level of αFP in 3 of them.

Three patients presented an increased level of βhCG in the CSF. It should be emphasized that all of them had elevated levels only in the CSF (with normal serum concentration). Discordant serum and CSF tumor marker results were observed and CSF analysis was needed for an accurate diagnosis, since protein levels are often much higher in the CSF than in the serum and more often exist in the CSF (40). Allen et al. demonstrated that approximately 34.5% out of 58 patients with histopathologically confirmed germinoma had elevated CSF βhCG values with normal serum values, and abnormal CSF values were higher than the serum value in 87% of patients with βhCG elevation (41).

Although serum and CSF βhCG, αFP, placental alkaline phosphatase – GCT markers – may be negative at the time of presentation in patients with CDI and PST, such markers may develop over time (40). However, it is worth remembering that pure germinomas typically present with normal βhCG and αFP levels in both the serum and CSF and the negative results of these biomarkers do not rule out the diagnosis which requires histological confirmation if suspected (42).

As regards MRI studies, the thickening of the hypophyseal stalk was the most common finding, together with the loss of neurohypophyseal hyperintensity in T1-weighted sequences, in which the lesion appeared isointense or slightly hyperintense with respect to the normal hypophysis. After gadolinium contrast injection, the uptake was less pronounced than in the normal hypophysis (43).

Imaging findings are not specific and the differential diagnosis must be established with lymphocytic hypophysis. Since this disorder is infrequent in childhood, histological findings compatible with a lymphocytic inflammatory process may represent the first sign of a host reaction to occult germinoma (29, 44, 45).

It would justify the determination of βhCG in the CSF in all prepubertal patients with a presumed or histological diagnosis of lymphocytic hypophysitis, as well as the immunohistochemical study of the histological specimen with the determination of placental lactogen, c-Kit and CD30 (46).

Germinoma should be suspected in all patients with PST and CDI, even when neurological and ophthalmological symptoms are absent. The course of germinoma is usually dramatic, with the tumor progressing rapidly, but not always. The diagnosis is usually made within 3 years, but, according to the literature, some cases were diagnosed even after 9 years (18, 47).

As regards our patients, the median time from the first symptoms to the diagnosis of germinoma was 2.69 years (IQR: 1.65-3.54).

Early diagnosis is the key to treat such tumors before the hypothalamic-hypophyseal damage proves irreversible, adjacent structures suffer compression, or a metastatic disease becomes apparent. The prognosis of the tumors is dependent upon the histology, but also upon the size of the tumor and the extent of the disease at the time of diagnosis.

Treatment includes chemotherapy and radiation therapy. The symptoms of hypopituitarism did not regress after the treatment in any of our patients.

LGG is rare in this location in children. It occurs most commonly in adults with no cases being described in children (48).

### Hypothalamo-Pituitary Histiocytosis

LCH, which we classified as a neoplastic disease, was the second most frequent cause of PST in our study. LCH underlying PST was diagnosed in 3 patients (13%). Two patients had M-LCH (previously diagnosed), and one had U-LCH in the hypothalamic-pituitary region (HPR). The first symptom of HPR LCH was CDI in all patients.

One patient aged 10 years and 5 months was diagnosed with U-LCH (HPR) confirmed with a biopsy. CDI and anterior pituitary deficiency (in terms of GH, TSH and ACTH) had been diagnosed one year earlier. The symptoms of CDI and hypopituitarism persisted.

M-LCH (lesions in the bones, lungs, lymph nodes and pituitary stalk) was diagnosed in a patient at 12 years of age. The diagnosis was made based on the history, clinical presentation and typical results of imaging tests (bone scintigraphy, HRCT of the lungs, PET/CT). The biopsy of the skin, skull lesions, lymph node, and bone marrow was inconclusive. Follow-up examination showed GH, LH/FSH deficiency and CDI in the patient. Another patient with M-LCH (lesions in the skin, bones, lungs, pituitary stalk) was diagnosed at 2 years and 2 months. Skin lesions preceded CDI by five months. The diagnosis was made based on the clinical presentation and imaging examinations (bone scintigraphy, HRCT, PET-CT), no biopsy was performed. GH deficiency treated with rhGH was still observed during follow up. CDI symptoms withdrew during treatment.

In both M-LCH cases, PST was normalized after oncological treatment.

LCH is a rare disease of the monocyte-macrophage system characterized by the clonal proliferation of the epidermal dendritic cells, which mainly affects the pediatric population with the annual incidence of approximately 6-10:100 000 and peaks between 1-3 years of age (49, 50). In recent years, increasing evidence has been published to support the idea that LCH is a “neoplasm”, which means that LCH tumors contain cells with gene mutations that cause them to make inappropriate copies of themselves. A specific gene called the *BRAF* is mutated in about half of LCH tumors (51).

Data from the literature also confirm that it is the second most common cause of PST/CDI in children (**Table 3**).

LCH is classified as unifocal LCH (solitary lesion), single-system multifocal disease (>1 lesions), multisystem LCH (≥2 organs/organ systems involved) and may affect any organ or system of the human body: the bones (78.7%), skin (36.7%), lung (25%), liver (25%), spleen (25%), hypothalamus and pituitary gland (25%), lymph nodes (15%) and other soft tissues (52, 53). In the CNS LCH exhibits a predilection for the HPR, leading to permanent posterior and/or anterior pituitary hormone deficiencies in a subset of patients. HPR infiltration is present in 5-50% of children with LCH, but most commonly in those with the multifocal disease form. Isolated HPR LCH is relatively rare (54). According to the French Langerhans’ Cell Histiocytosis Study Group database, isolated CDI was the initial presentation of LCH in 26 patients and the pituitary stalk was enlarged in 14 of 1135 LCH patients under 18 years of age (55).

In a large series of patients with LCH (1242 study subjects), only 0.04-0.6% had isolated HPR involvement (56). Isolated LCH of the infundibulum is considerably rare, and such instances are typically reported as singular cases in the literature (57). CDI, the most frequent hormonal abnormality, occurs in 15-50% of patients with HPR LCH (54). It may occur before, concurrently with, or many years after other multisystem manifestations of the disease that leads to the diagnosis of histiocytosis.

Pituitary dysfunction occurs in only 5-20% of patients. It is almost always accompanied by CDI (94%). The most common types of hormone deficiency in children and adults with HPR involved LCH include growth hormone (GH) deficiency (53-67%), followed by gonadotropin (53-58%), and thyroid-stimulating hormone (3.9%) deficiencies. Adrenocortical hormone deficiency is observed in 1-2% and hyperprolactinemia in 40% (58).

**Figure 7:**
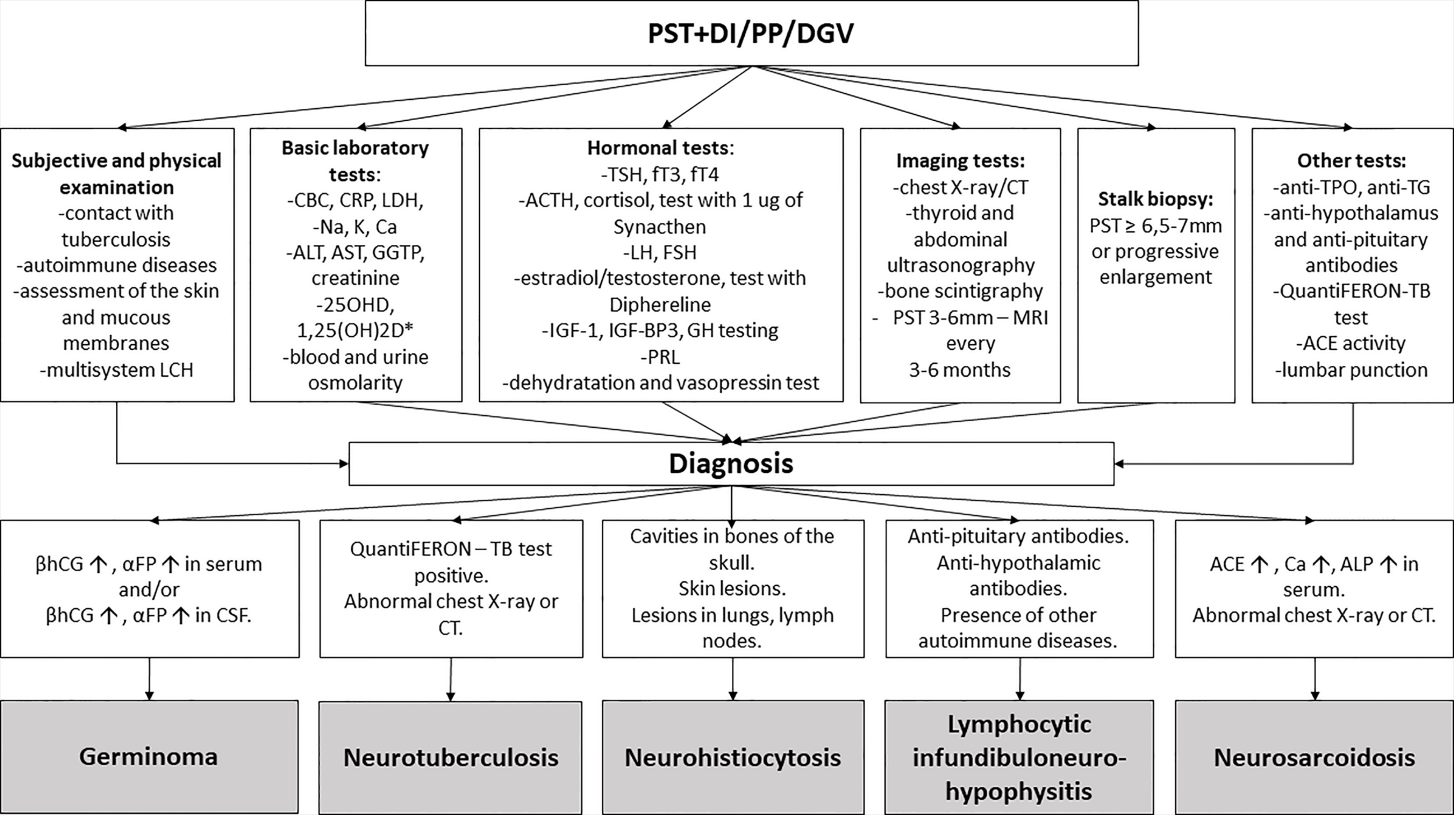
The diagnostic process of pituitary stalk thickening. PST, pituitary stalk thickening; DI, diabetes insipidus; PP, precocious puberty; DGV, decreased growth velocity; LCH, Langerhans cell histiocytosis; CBC, complete blood count; CRP, c-reactive protein; LDH lactate dehydrogenase; ALAT, alanine transaminase; ASPAT, aspartate transaminase; GGTP, gamma-glutamyl transpeptidase; 1,250HD, 1,25-dihydroxyvitamin D; 250HD, 25-hydroxycholecalciferol; Ca, calcium; Na, sodium; K, potassium; TSH, thyroid-stimulating hormone; FT3, free triiodothyronine; FT4, free thyroxine; ACTH, adrenocorticotropic hormone; LH, luteinizing hormone; FSH, follicle-stimulating hormone; IGF-1, insulin-like growth factor 1; IGF-BP3, insulin-like growth factor-binding protein 3; GH, growth hormone; PRL, prolactin, CT- computed tomography; anti-TPO, thyroid peroxidase antibody; anti-TG, antithyroglobulin antibody; ACE, angiotensin-converting enzyme. TB, tuberculosis, βhCG- beta subunits of human chorionic gonadotropin; αFP- alpha-fetoprotein; ALP, alkaline phosphatase; MRI, magnetic resonance imaging; CSF, cerebrospinal fluid. * in case of suspicion of sarcoidosis.

Currently, there is no specific biological marker of disease activity. Tests performed when LCH is suspected include: dermatological survey, laboratory evaluation (complete blood count, blood chemistry tests) and radiographic evaluation (skeletal radiography survey, bone scintigraphy, chest radiograph and ultrasonography of the abdomen) to confirm other LCH lesions (**Figure 7**).

As regards HPR LCH, an MRI scan typically shows an enlargement of the pituitary stalk enhanced after gadolinium injection and the loss of spontaneous T1 hyperintensity of the posterior pituitary.

It is still difficult to differentiate HPR involved LCH from other diseases, such as germinoma or lymphocytic hypophysitis with the use of MRI. An isolated HPR lesion is a clinical challenge, because neither the clinical manifestations nor MRI can facilitate making a definitive diagnosis, and the risk associated with obtaining a diagnostic biopsy remains high. PST diagnostic difficulties are presented in an article by Nagasaki et al. They described a 13-year-old girl with CDI and PST. The patient had LCH which was confirmed in a biopsy. Her CSF βhCG was slightly elevated and a preliminary diagnosis of germinoma was made (57).

The gold standard for LCH includes positive histology and immunohistochemistry evidence – the presence of Birbeck granules under electron microscopy, positive immunohistochemical staining for the protein markers S100 and CD1and/or CD 207 (Langerin) (59).

The optimal treatment of HPR LCH remains challenging to define because of the very low incidence of disease and a paucity of prospective studies. According to the consensus recommendation for treatment of LCH, simple observation, surgery, low-dose radiation and chemotherapy are considered in the treatment planning (53). Limited literature data showed that low-dose irradiation (≤22 Gy) was usually the first-line therapy adequate for most cases of isolated HPR LCH with a sporadic recurrence of illness (60).

Spontaneous regression was well reported in numerous body systems including the skin, bone, lungs and the CNS (pituitary stalk) (57, 61).

No spontaneous remission of the disease was observed in our study. PST was normalized in two cases after chemotherapy (steroids, methotrexate, vinblastine, cladribine). Moreover, a surgery was performed (total removal of the lesion) in one patient with the HPR-localized form.

### Lymphocytic Hypophysitis

No case of lymphocytic hypophysitis (LYH) was histopathologically confirmed in our study. Immunological etiology was considered in one patient (No 21). The patient, aged 13 years and seven months, presented with symptoms of CDI, headaches and vomiting. In the laboratory tests, antibodies against the pituitary gland and anti-hypothalamus were present. Evaluation of the anterior pituitary function confirmed no abnormalities. MRI revealed PST of 4 mm and invisible posterior pituitary lobe. Repeat MR scans showed that pituitary stalk lesion resolved, making an inflammatory lesion the most likely etiology. CDI and invisible posterior pituitary lobe persist in follow-up. Granulomatous disease screen was negative and she had no symptoms consistent with those pathologies. Such cases of spontaneous regression were reported by other authors in the literature and were related to sarcoidosis, LCH and LYH (62).

LYH is a neuroendocrine disorder characterized by the autoimmune inflammation of the pituitary gland with various degrees of pituitary dysfunction.

It was initially considered to be restricted to women in relation to pregnancy, but it is now clear that LYH may occur in children and adults, males and females (63).

The following types of LYH may be distinguished: lymphocytic adenohypophysis (LAH – an inflammation limited to the anterior hypophysis), lymphocytic infundibuloneurohypophysitis (LINH – autoimmune infiltration involving the infundibular stem and posterior lobe), and lymphocytic panhypophysitis (LPH – both the adenohypophysis and the infundibuloneurohypophysis are affected) depending on which part of the HPR is involved in the immune process.

Although LINH was first described in 1970 by Sito et al. in a 66-year-old woman with CDI, it was most commonly reported in children and adolescents (64).

The annual incidence of LYH may be estimated at one case per 9 million. Data including 4 large patient analyses (905–2500) indicated that LYH was seen in less than 1% (0.88-0.24%) of all surgical pituitary specimens (65–67). In 2012, Kalra et al. indicated that the number of children under 18 with LYH was greater than 96 in the literature (68). The incidence in children was much lower compared to approximately 460 adults with autoimmune hypophysitis (69). There was no significant sex predilection among children. In adults, more female patients were identified with the female to male ratio of 8:1 (70).

Histopathology remains the gold standard for the diagnosis of LYH. The defining pathological feature of LYH is the infiltration of the pituitary gland with lymphocytes, with the predominance of T cells and particular CD4 cells. Plasma cells, eosinophils, macrophages, histiocytes, and neutrophils are also present. Fibrosis and rare necrosis may be reported in pathological specimens (71).

Concomitant autoimmune conditions were reported in 20-50% of cases of LYH (72, 73). Autoimmune thyroid disease was the most common association. It was reported in 15-25% of LYH cases, i.e., 70-80% of cases with an associated autoimmune disease. Chronic autoimmune thyroiditis constituted about 75%, while Graves’ disease and subacute thyroiditis were reported less frequently. Autoimmune adrenalitis was reported in 5-7% of cases (15-25% of patients with an associated autoimmune condition), while pernicious anemia and type 1 diabetes (T1D) were seen in 2% of cases (73). Associated autoimmune disorders were seen in 7 of 96 children with the incidence of 7% compared to 20-50% in adults (68).

The clinical presentation of LYH is variable and comprises four categories of symptoms: sellar compression, hypopituitarism, CDI, and hyperprolactinemia.

The symptoms of sellar compression are rare in LINH. Headache affects 13% of patients and visual abnormalities include visual field defects, decreased acuity and diplopia in only 3% of patients (74).

Other most common symptoms occur due to deficiency of anterior pituitary hormones (hypocortisolism, hypothyroidism, hypogonadism). CDI is another most common issue, which may be attributed either to direct immune destruction or to the compression of the posterior lobe and infundibular stem. CDI is the cardinal feature of LINH (98%). CDI may be masked in the presence of a concomitant glucocorticoid deficit, because they oppose the action of antidiuretic hormone (ADH) at several levels. Glucocorticoids inhibit the secretion of ADH from the neurons of the paraventricular nucleus and suppress the synthesis of aquaporin 2, an ADH-dependent water channel expressed in the collecting tubule of the kidneys (75). The manifestations of hyperprolactinemia are the least common. They are mainly represented by amenorrhea and galactorrhea (72).

Antibodies against pituitary protein and vasopressin-secreting hypothalamic cells may be found in patients with LYH. The specificity and sensitivity of pituitary antibodies are poor, as they are found in various pituitary diseases such as Cushing’s disease, pituitary adenoma, empty sella syndrome, as well as in other autoimmune diseases such as T1D, Hashimoto’s thyroiditis, and Graves’ disease (76). A retrospective analysis was conducted by Caturelli et al. and included 379 patients with LYH. Pituitary antibodies were detected in 10-80% of the patients depending on the form of LYH and the method used, more often in immunoblotting (76). Additionally, data of Chiloiro et al. suggest that the study of HLA haplotypes, particularly the DQ8 ones, may play an important role in diagnosis of LYH, as HLA haplotypes indicate a genetic predisposition to the associated autoimmune disease (77).

In our study, antibodies against the pituitary gland and anti-hypothalamus were tested in four patients. The results were positive in three of them. One patient was diagnosed with GCT, the second with suspicion of LINH, another is still under observation for LGG.

As regards MRI in patients with LINH, diffuse PST is very characteristic, with a greater diameter exceeding 3.5 mm at the level of the median eminence of the hypothalamus (78). The normal smooth tapering of the infundibular stalk is lost and a varying degree of asymmetry may occur. Marked gadolinium enhancement of the stalk is quite common, extending even into the lower hypothalamus. The loss of the usual neurohypophyseal “bright spot” was also commonly reported. However, it should be noted that this MRI sign may be absent in 10% of normal subjects (71).

A presumptive diagnosis of LYH may be made based on clinical, laboratory, and imaging studies, but the definitive confirmation requires a pituitary biopsy.

The clinician should suspect LINH in patients with the acute onset of CDI with headache and mass-effect symptoms; with an isolated, early or disproportionate disruption of ACTH or TSH secretion, disproportionate disruption of anterior pituitary function for the magnitude of the changes on MR imaging; the presence of other autoimmune conditions and/or positive autoantibodies, the presence of anti-pituitary/anti-hypothalamus antibodies in the serum; lymphocytic pleocytosis in the CSF; characteristic MRI findings (diffuse thickening of the pituitary stalk with or without enhancement after gadolinium, loss of the normal posterior “bright spot” on T1-weighted images) (79) (**Figure 7**).

The treatment of LYH is currently only symptomatic. It includes reducing the size of the pituitary mass and/or replacing the defective endocrine function. Mass reduction may be achieved *via* pituitary surgery, lympholytic drugs, or radiotherapy.

Glucocorticoids should be used as the first line of treatment with the most commonly used ones being prednisone, hydrocortisone, and methylprednisolone (78, 80, 81). Other immunosuppressive drugs, such as azathioprine, methotrexate, or cyclosporine A may be used in patients with poor response to glucocorticoids (67, 82).

Surgery should be performed only in the presence of serious and progressive deficits of the visual field, visual acuity, ocular movements, or increased intracranial pressure, not responding to pharmacological treatment.

Radiotherapy (conventional fractionated external-beam radiotherapy, 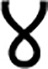-knife radiosurgery) should be reserved for cases with severe mass effect symptoms, which show a poor response or are poor candidates for high doses of glucocorticosteroids and/or surgery. However, it should be remembered that, as reported by Caturegli et al., LYH resolved spontaneously without any treatment in 11 patients (3%) (76).

### Hypothalamic-Pituitary Sarcoidosis

Neurosarcoidosis was not diagnosed in any of the patients, probably due to the rare occurrence of this disease in children, as it mainly develops in patients aged 45-55.

Despite its casuistic incidence, neurosarcoidosis should be considered in the differential diagnosis.

Sarcoidosis is an auto-inflammatory, granulomatous disorder of unknown etiology, typically manifesting with multi-organ involvement. Hilar lymphadenopathy and pulmonary interstitial infiltration are the most common manifestations, but the pathology may also affect the eyes, skin, liver, and spleen (83). The incidence of clinically recognized sarcoidosis in children is 0.22-0.29:100 000 children per year, with a small peak at 13-15 years of age (84). Neurosarcoidosis was reported in 5-10% of adults with systemic sarcoidosis and was seldom recognized in children (85). According to the literature published in English, 54 children and adolescents were reported to be diagnosed with neurosarcoidosis, and only nine children with isolated neurosarcoidosis. The average age at onset was 12 years (3 months – 18 years) (86, 87). Neurosarcoidosis may affect: the meninges, ventricles, cerebellar hemisphere, the white matter of the frontal lobe, spinal cord parenchyma, optic nerve and chiasm, other cranial nerves (facial, auditory, vestibulocochlear) and the pituitary stalk, pituitary, thalamus, and hypothalamus.

The manifestations of neurosarcoidosis include seizures, headache, vomiting, somnolence, cranial neuropathies (common: VII, II, rare: III, IV, VI, V, VIII), hypothalamic dysfunction, cerebellar signs, neuropsychological deficits, myelopathy, and peripheral neuropathy. Granulomatous lesions that affect the hypothalamus and pituitary gland cause CDI, growth and sexual maturation failure, syndrome of inappropriate antidiuretic hormone secretion (SIADH). The frequency of hypothalamic-pituitary disturbances is estimated at 6-9% of neurosarcoidosis (88).

Kidd et al. described that out of 6 patients with the hypothalamic involvement of sarcoidosis only 2 had an enlargement of the stalk (89).

There is no single diagnostic test for sarcoidosis. Neurosarcoidosis is a diagnosis of exclusion. Diagnosis is achieved by the combination of history, examination, serum/CSF ACE, brain MRI, CSF analysis, gallium-67 scintigraphy and biopsy. Biopsy still provides the strongest evidence for this disease (**Figure 7**).

Serum ACE levels were checked in 6 of our patients, 3 of whom had elevated concentrations (two patients were finally diagnosed with germinoma, and one was monitored for LGG). ACE levels are increased in sarcoidosis because of the activation of monocytes, which are the precursors to the epithelioid cells that form granulomas (90). ACE is not specific for sarcoidosis, elevated serum ACE is also observed in tuberculosis, lung cancer, Hodgkin lymphoma, and cirrhosis of the liver. Increased serum level is confirmed in 68-88% of patients with sarcoidosis and the CSF concentration in 24-55% of patients with neurosarcoidosis (91).

Normal serum/CSF ACE level cannot exclude the diagnosis of sarcoidosis.

In patients with neurosarcoidosis the analysis of CSF may show pleocytosis (>5 white blood cells) with slight lymphocytosis (10-100 cells/uL), slightly elevated protein (>50 mg/dl), slightly decreased glucose (<50 mg/dl) and increased immunoglobulins with oligoclonal banding (88). However, CSF abnormalities are not specific to neurosarcoidosis and normal CSF results were obtained in over a third of cases (92). A retrospective review of the Mayo Clinic record system revealed that oligoclonal banding was present in the spinal fluid of 18% of patients with neurosarcoidosis (93).

Anemia, leukopenia, and eosinophilia are commonly seen in blood counts (94).

Hypercalcemia and/or hypercalciuria occur in up to 10 and 40% of children, respectively, because sarcoid macrophage is able to synthesize 1,25-dihydroxyvitamin D (95). Corticosteroids are widely used and are often effective in the treatment of sarcoidosis. If corticosteroid therapy fails, second-line agents, like methotrexate, azathioprine, mycophenolate mofetil, leflunomide or third-line agents like tumor necrosis factor inhibitors – infliximab or adalimumab, are indicated in the treatment (96, 97).

### Tubercular Hypophysitis

Pituitary tuberculosis was not diagnosed in any of our patients. Even though Poland is not an endemic country, we considered this pathology in the differential diagnosis. None of the patients had a history of contact with tuberculosis. QuantiFERON test was performed in 8 patients with PST and negative results were obtained. Similarly, chest X-ray/CT scan performed in several patients did not reveal any lesions typical of tuberculosis.

The incidence of tubercular hypophysitis is 0.5-4% of all intracranial lesions and 25-30% of tubercular hypophysitis cases reported in literature had previous or active tuberculosis (98). Primary pituitary tuberculosis caused by Mycobacterium tuberculosis, is a sporadic condition, and only 106 cases were reported from 1924 till 2019 (99). Tuberculosis usually occurs in endemic countries and it is rare in children. Headache, somnolence, visual disturbances, low-grade fever and vomiting are common clinical symptoms (100). Anterior pituitary insufficiency, hyperprolactinemia and CDI are common endocrine dysfunctions in pituitary tuberculosis (101). In MRI, pituitary tuberculosis presents with thickening and nodularity of the pituitary stalk, the sellar and suprasellar mass, which can be difficult to differentiate from adenoma (100). Diagnostic tests include: tuberculin skin test, QuantiFERON-TB test (sensitivity 92.6%), chest X-ray/CT (**Figure 7**). Histopathological examination reveals a necrotizing granulomatous inflammation, with acid-fast bacilli stains (usually not demonstrable from the pathological tissue or CSF), a polymerase chain reaction of *Mycobacterium* spp. in DNA extracted from the tissue/CSF. A delayed diagnosis might lead to permanent endocrine dysfunction. Surgery is not indicated in tuberculous hypophysitis except for obtaining biopsies to confirm the diagnosis. Antitubercular drugs that cross the blood-brain barrier are given to patients for 9-24 months depending on the clinical and imaging outcome (100, 101). Early diagnosis is very important in cases of tubercular hypophysitis, given that panhypopituitarism may be completely eliminated in clinical terms following effective anti-tuberculosis treatment (102).

### Diagnostics

Pituitary stalk biopsy was performed in 2 (8.7%) of our patients. This procedure is rarely performed. Only 24-40% of patients with PST undergo this procedure (3, 4, 9). According to the UK consensus guideline (2021), children who continue to pose a diagnostic dilemma after appropriate serial neuroimaging should undergo whole-body imaging. After repeat CSF testing, a biopsy of the PST should be considered in case of very large (≥6.5-7 mm) or progressively enlarging stalk, evolving hypopituitarism, visual deterioration, or a combination of all three factors, as they are more likely to suggest a neoplasm than mild, isolated PST (11, 103). Other authors also recommend biopsies only for lesions larger than 7 mm, and in other cases repeated cerebral MRI and testing tumor markers in the serum and CSF should be performed (18). Biopsy should only be done by a skilled pituitary surgeon. The following surgery accesses are possible: transsphenoidal, endoscopic, and the base of skull techniques. Preoperative and postoperative pediatric endocrine support on-site is essential, hence the need for multi-professional, specialist center care (103). Endoscopic transsphenoidal biopsy seems to be safe and does not cause any endocrinological or neurological complications (3). Regrettably, it is not feasible in younger children due to the development of the nasal sinuses (104). Endoscope-assisted microsurgery *via* the supraorbital keyhole approach is also a low-risk method to obtain biopsy material, also in the youngest patients (105). Furthermore, a biopsy may not provide a clear diagnosis. Germinoma may mimic inflammatory processes due to lymphocytic infiltration surrounding the tumor which may lead to misdiagnosis and the delay of proper treatment (44).

Due to the lack of histopathological data, MRI remains the “gold standard” in pituitary stalk thickening diagnostics. The appearance of the pituitary stalk may give many diagnostic clues. The concomitant involvement of the pineal gland or basal ganglia is highly suggestive of germinoma and should involve an additional MRI examination of the spine (106). Brain MRI may also reveal soft tissue or skull lesions suggestive of Langerhans cell histiocytosis (106). Turcu et al. postulated that specific patterns of gadolinium enhancement on head MRI might suggest congenital lesions (3). Sbardella et al. suggested that the textural analysis of MRI performed with specific software might help differentiate neoplastic and non-neoplastic lesions based on grey levels in the region of interest. Comparing neoplastic and non-neoplastic disorders, texture analysis revealed a significantly higher degree of heterogeneity expressed in patients with neoplastic lesions compared to non-neoplastic lesions (107).

According to different authors repeated brain MRI and the investigation of the tumor marker βhCG in the serum and CSF should be performed every 3-6 months during the first 3 years after the onset of PST/CDI, in order to rapidly establish a diagnosis before the developed large tumor leads to visual and neurological symptoms, because neoplastic disorders are most likely to develop during this period. MRI should be performed every year during the following 2 years and every 2-5 years thereafter, depending on the size and evolution of the lesion (108). This procedure is recommended because, occasionally, occult LCH was detected up to 10 years after the presentation and germ cell tumor up to 20 years afterwards (106, 109, 110). Therefore, the follow-up strategy should be individually adapted.

74% of our patients received the final diagnosis within three years, consistently with other authors’ reports (18, 47). However, in our clinic, we observed one case of germinoma which became apparent after almost 5 years of observation. Another patient was observed for 4.5 years and LGG was suspected.

Both neurosurgery procedures and radiotherapy pose a risk of pituitary damage and chronic hormone deficiencies.

Patients were treated with replacement therapy for diagnosed hormonal deficits.

GH therapy was introduced in eleven patients, ten with germinoma, one with LCH. In accordance with Pediatric Endocrinology Society guidelines, we waited until 12 months with no evidence of ongoing tumor after completion of tumor therapy had passed before initiating GH treatment (111). The GH treatment was effective in increasing height in our patients. Similary to meta-analyzes of GH therapy in childhood cancer survivors, no increased risk of recurrence or secondary neoplasm was observed (112). It is also important that patients are transitioned to adult care after reaching the age of majority, where hormonal treatment is continued.

Therefore, due to numerous metabolic and neurological complications after oncological treatment patients still require constant care of a neurologist, oncologist, gynecologist, ophthalmologist and diabetologist.

Based on our own experience and data from the literature, the authors proposed a diagnostic diagram of PST in children (**Figure 7**).

## Conclusions

The spectrum of pathology involving the pituitary infundibulum is broad. The pituitary infundibulum presents a diagnostic imaging challenge because of its small size and the protean spectrum of disease processes.

Our study revealed that CNS tumors, germinoma in particular, are the most common causes of PST in children. Histiocytosis or LYH were rarely diagnosed. Neither neurosarcoidosis nor specific inflammation were found in our study group, which seems to be related to the inclusion of patients under 18 years of age. Germinoma should be suspected in all children with PST, especially with CDI, even if neurological and ophthalmological symptoms are absent. For quick diagnosis and implementation of the treatment, it is crucial that radiologists and clinicians are familiar with the characteristics of GCTs.

Despite the overall survival rate during the observational period in patients diagnosed with germinoma being favorable (88.2%), the quality of life assessed in adulthood was reduced. The main health problems were obesity, metabolic syndrome, type 2 diabetes, hypopituitarism (mainly in terms of GH, TSH, ACTH), and profound hearing loss requiring hearing aids. It is worth noting that none of the adult patients was employed, which indicates the need for professional and social activation of this group of patients. It is essential to diagnose and introduce treatment as soon as possible in order to reduce the morbidity in this group of patients and improve their quality of life.

## Study Limitations

Our study has several limitations. First of all, the patient group is small, so we could not present clear conclusions and recommendations for patients with this disorder. However, the group included all patients treated in our Department in the years 1990-2021. Secondly, it is a retrospective study. Thirdly, we only analyzed the pediatric population. We take care of patients up to 18 years of age and it is impossible to establish contact with all adult patients. Information about the health of adult patients was collected orally or by a letter from the patients themselves. The authors did not receive medical documentation, so information about long-term complications, tumor recurrence or fertility might be incomplete.

## Data Availability Statement

The original contributions presented in the study are included in the article/supplementary material. Further inquiries can be directed to the corresponding author.

## Ethics Statement

Ethical review and approval was not required for the study on human participants in accordance with the local legislation and institutional requirements. Written informed consent to participate in this study was provided by the participants’ legal guardian/next of kin. Written informed consent was obtained from the individual(s), and minor(s)’ legal guardian/next of kin, for the publication of any potentially identifiable images or data included in this article.

## Author Contributions

Design and conception: EM. Data collection and analyses: EM, KK, MP-P, DM, and WG. Writing of the manuscript: EM and KK. Editing and reviewing the manuscript: EM and MB-W. All authors contributed to the article and approved the submitted version.

## Conflict of Interest

The authors declare that the research was conducted in the absence of any commercial or financial relationships that could be construed as a potential conflict of interest.

## Publisher’s Note

All claims expressed in this article are solely those of the authors and do not necessarily represent those of their affiliated organizations, or those of the publisher, the editors and the reviewers. Any product that may be evaluated in this article, or claim that may be made by its manufacturer, is not guaranteed or endorsed by the publisher.

## Glossary

**Table:**

ALP	alkaline phosphatase
ACE	angiotensin-converting enzyme
ACTH	Adrenocorticotropic hormone
ALAT	alanine transaminase
ASPAT	aspartate transaminase
αFP	alfa-fetoprotein
βhCG	beta-human chorionic gonadotropin
Ca	calcium
CBC	complete blood count
CDI	central diabetes insipidus
CMHI	Children’s Memorial Health Institute
CNS	central nervous system
CRP	c-reactive protein
CSF	cerebrospinal fluid
DGV	decreased growth velocity
F	female
^18^F-FDG PET/CT	^18^F-fluorodeoxyglucose positron emission tomography/computed tomography
FSH	follicle-stimulating hormone
FT3	free triiodothyronine
FT4	free thyroxine
GGTP	gamma-glutamyl transpeptidase
GH	growth hormone
GHD	growth hormone deficiency
GnRH	gonadotropin-releasing hormone
GCT	germ cell tumor
hCG	human chorionic gonadotropin
HPR	hypothalamic-pituitary region
HRCT	high - resolution computed tomography
HV	headache and vomiting
IGF-1	insulin-like growth factor 1
IGF-BP3	insulin-like growth factor-binding protein 3
IgA	immunoglobulinA
IgM	immunoglobulin M
K	potassium
LCH	Langerhans cell histiocytosis
LDH	lactate dehydrogenase
LGG	low-grade glioma
LINH	lymphocytic infundibuloneurohypophysitis
LH	luteinizing hormone
M	male
MRI	magnetic resonance imaging
Na	sodium
NGGCTs	non-germinomatous germ cell tumors
N	normal range
OS	overall survival
PLAP	placental alkaline phosphatase
PP	precocious puberty
PRL	prolactin
PST	pituitary stalk thickening
SDV	sudden deterioration in vision
anti-Tg	antithyroglobulin antibody
anti-TPO	thyroid peroxidase antibody
TSH	thyroid-stimulating hormone
T2D	type 2 diabetes
rhGH	recombinant human growth hormone
wnr	within normal range
1,25OHD	1,25-dihydroxyvitamin D
25OHD	25-hydroxycholecalciferol
